# The LEAD study protocol: a mixed-method cohort study evaluating the lung cancer diagnostic and pre-treatment pathways of patients from Culturally and Linguistically Diverse (CALD) backgrounds compared to patients from Anglo-Australian backgrounds

**DOI:** 10.1186/s12885-018-4671-4

**Published:** 2018-07-21

**Authors:** Danielle Mazza, Xiaoping Lin, Fiona M. Walter, Jane M. Young, David J. Barnes, Paul Mitchell, Bianca Brijnath, Andrew Martin, Jon D. Emery

**Affiliations:** 1 Department of General Practice, Monash University, Building 1, 270 Ferntree Gully Road, Notting Hill, Victoria 3168 Australia; 2 The Primary Care Unit, Department of Public Health and Primary Care, University of Cambridge, Cambridge, UK; 3 Sydney School of Public Health, Sydney Medical School, University of Sydney, Camperdown, Australia; 4 Department of Respiratory and Sleep Medicine, Royal Prince Alfred Hospital, Camperdown, Australia; 5 Olivia Newton-John Cancer and Wellness Centre, Austin Health, Heidelberg, Australia; 6 University of Melbourne, Parkville, Australia; 7 Social Gerontology Division, National Ageing Research Institute, Parkville, Australia; 8 NHMRC Clinical Trials Centre, University of Sydney, Camperdown, Australia; 9 Department of General Practice and Centre for Cancer Research, Faculty of Medicine, Dentistry and Health Sciences, University of Melbourne, Carlton, Australia

**Keywords:** Lung cancer, Ethnicity, Time intervals, Cancer diagnostic pathway

## Abstract

**Background:**

Lung cancer is the leading cause of cancer mortality worldwide. Early diagnosis and treatment is a key factor in reducing mortality and improving patient outcomes. To achieve this, it is important to understand the diagnostic pathways of cancer patients. Patients from Culturally and Linguistically Diverse (CALD) are a vulnerable group for lung cancer with higher mortality rates than Caucasian patients. The aim of this study is to explore differences in the lung cancer diagnostic pathways between CALD and Anglo-Australian patients and factors underlying these differences.

**Methods:**

This is a prospective, observational cohort study using a mixed-method approach. Quantitative data regarding time intervals in the lung cancer diagnostic pathways will be gathered via patient surveys, General practitioner (GP) review of general practice records, and case-note analysis of hospital records. Qualitative data will be gathered via structured interviews with lung cancer patients, GPs, and hospital specialists. The study will be conducted in five study sites across three states in Australia. Anglo-Australian patients and patients from five CALD groups (i.e., Arabic, Chinese, Greek, Italian and Vietnamese communities) will mainly be identified through the list of new cases presented at lung multidisciplinary team meetings. For the quantitative component, it is anticipated that 724 patients (362 Anglo-Australian and 362 CALD patients) will be recruited to obtain a final sample of 290 (145 per group) assuming a 50% patient survey completion rate and a 80% GP record review completion rate. For the qualitative component, 60 interviews with lung cancer patients (10 Anglo-Australian and 10 patients per CALD group), 20 interviews with GPs, and 20 interviews with specialists will be conducted.

**Discussion:**

This is the first Australian study to compare the time intervals along the lung cancer diagnostic pathway between CALD and Anglo-Australian patients. The study will also explore the underlying patient, healthcare provider, and health system factors that influence the time intervals in the two groups. This information will improve our understanding of the effect of ethnicity on health outcomes among lung cancer patients and will inform future interventions aimed at early diagnosis and treatment for lung cancer, particularly patients from CALD backgrounds.

**Trial registration:**

The project was retrospectively registered with Australian New Zealand Clinical Trials Registry (registration number: ACTRN12617000957392, date registered: 4th July 2017).

## Background

Lung cancer is the most common cancer worldwide. In 2012, it was estimated that there were 1.8 million new cases of lung cancer, accounting for 13% of all incident cancer cases [1]. Lung cancer is also the leading cause of cancer mortality, estimated to be responsible for 1.59 million (or 19.4% of the total) cancer deaths in 2012 [1]. One reason for this high mortality rate is that lung cancer is often diagnosed at a late stage, which is associated with higher mortality than early-stage disease [2–5]. Walters et al. (2013) analysed population-based data of lung cancer between 2004 and 2007 in six developed countries (including Australia, Canada, Denmark, Norway, Sweden and the UK) and found that at least half of lung cancer patients were diagnosed at a late stage when curative treatment is unlikely as an option.

Early diagnosis and treatment is considered a key factor in reducing lung cancer mortality and improving patient outcomes [6]. When cancer patients are diagnosed early, they are more likely to be suitable for curative treatment, leading to a greater probability of survival, less morbidity, and improved quality of life [7]. To achieve early diagnosis and treatment, it is important to understand the diagnostic and treatment pathways of cancer patients in order to inform the development of interventions to reduce diagnostic and treatment delay [6, 8]. Guided by the Model of Pathways to Treatment [8], the LEAD project (Lung cancer diagnostic and treatment pathways: a comparison between Culturally and linguistically diverse [CALD] and Anglo-Australian patients) will use a mixed-method, observational cohort design to explore the pathways to diagnosis and pre-treatment of lung cancer patients in multicultural Australia.

### Lung cancer among the CALD population

The LEAD project focuses on these differences, because there is evidence suggesting that compared to Caucasian lung cancer patients, CALD patients are a vulnerable group, with poorer survival rates and a lower likelihood of receiving timely and appropriate treatment [9–12]. Possible reasons for these poorer outcomes include more advanced stage at diagnosis, cultural beliefs towards treatment, fatalism and medical mistrust [10, 13, 14].

Similar to many Western countries, people from CALD backgrounds account for a significant proportion of Australia’s population. Data from the most recent census shows that in 2016, Australia’s population consisted of people from over 300 ethnic groups with more than a quarter (26%) born overseas and a further one-fifth (20%) having at least one overseas-born parent [15]. This cultural diversity has been reflected in lung cancer patients. A recent retrospective cohort study with six public and two private hospitals in Victoria Australia found that, of the 1417 patients diagnosed with lung cancer between 2011 and 2014, 51% were born overseas [16].

However, most of current research with the CALD population has been conducted in the United States (US). Given the significant differences in the healthcare system and the composition of the CALD communities between Australia and the US (for example, the top three countries of birth for the overseas-born population in 2016 were England, New Zealand, and China for Australia [Australian Bureau of Statistics, 2017], and Mexico, China and India for the US [Migration Policy Institute, 2018]), it is important to explore whether the finding of poorer outcomes among CALD lung cancer patients in the US studies also applies to the Australian context.

### Model of Pathways to Treatment

The diagnostic and treatment pathway of lung cancer patients is complex, comprising multiple stages from patients noticing symptoms and seeking help from health professionals, to obtaining a formal diagnosis and starting treatment [6]. It is, therefore, useful to apply a theoretical model in cancer pathway studies to inform the description and measurement of the stages along this pathway [6].

**Fig. 1:**
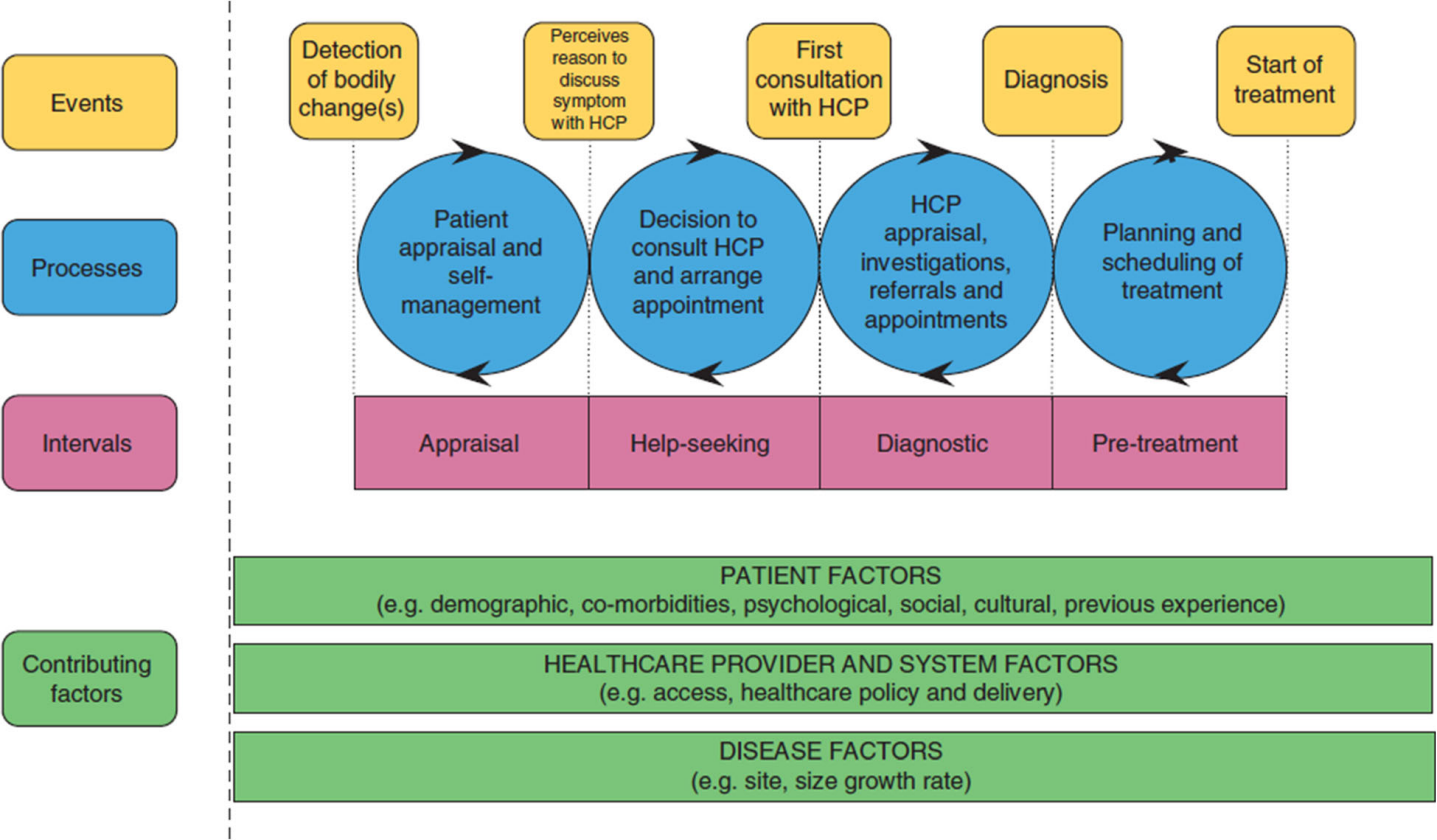
Model of Pathways to Treatment [8]. HCP: health care provider

The Model of Pathways to Treatment [8] will be used in the LEAD project as the theoretical model to understand and measure the cancer diagnostic and pre-treatment pathway. This model is built on the findings from a systematic review and has been incorporated into the Aarhus Statement, an international guideline for the design and reporting of studies on early cancer diagnosis [6]. An important feature of this model is that it uses events that can be readily understood by patients, clinicians and researchers to define the key time intervals underlying this pathway [8]. As shown in Fig. 1, these intervals are: (1) the appraisal interval (time between first detection or awareness of a symptom to recognising a need to discuss the symptom with a healthcare professional), (2) the help-seeking interval (time from recognising the need to discuss their symptoms to attending the first consultation with a healthcare professional); (3) the diagnostic interval (time from first consultation to a formal cancer diagnosis), and (4) the pre-treatment interval (time from the formal diagnosis to initiation of treatment) [8].

Another important feature of this model is that it considers and categorises factors that are likely to have important impacts on these time intervals. These include: (1) patient factors (e.g. demographic, co-morbidities, and cultural factors), (2) healthcare provider and system factors (e.g. access, healthcare policy), and (3) disease factors (e.g., site, size) [8]. This framework facilitates a systematic investigation of the enablers and barriers that are encountered along the cancer diagnostic and pre-treatment pathway.

### Study aims

The two aims of the LEAD project are (1) to explore the differences in the four time intervals along the lung cancer diagnostic pathway between CALD and Anglo-Australian patients, and (2) to explore patient, health care provider, and health system factors that are associated with the differences in time intervals between the two groups. Based on earlier studies, we hypothesise that CALD patients will report longer time intervals than Anglo-Australian patients. There is no specific hypothesis associated with the second research aim because it is exploratory in nature.

## Methods/Design

### Study design and setting

**Fig. 2:**
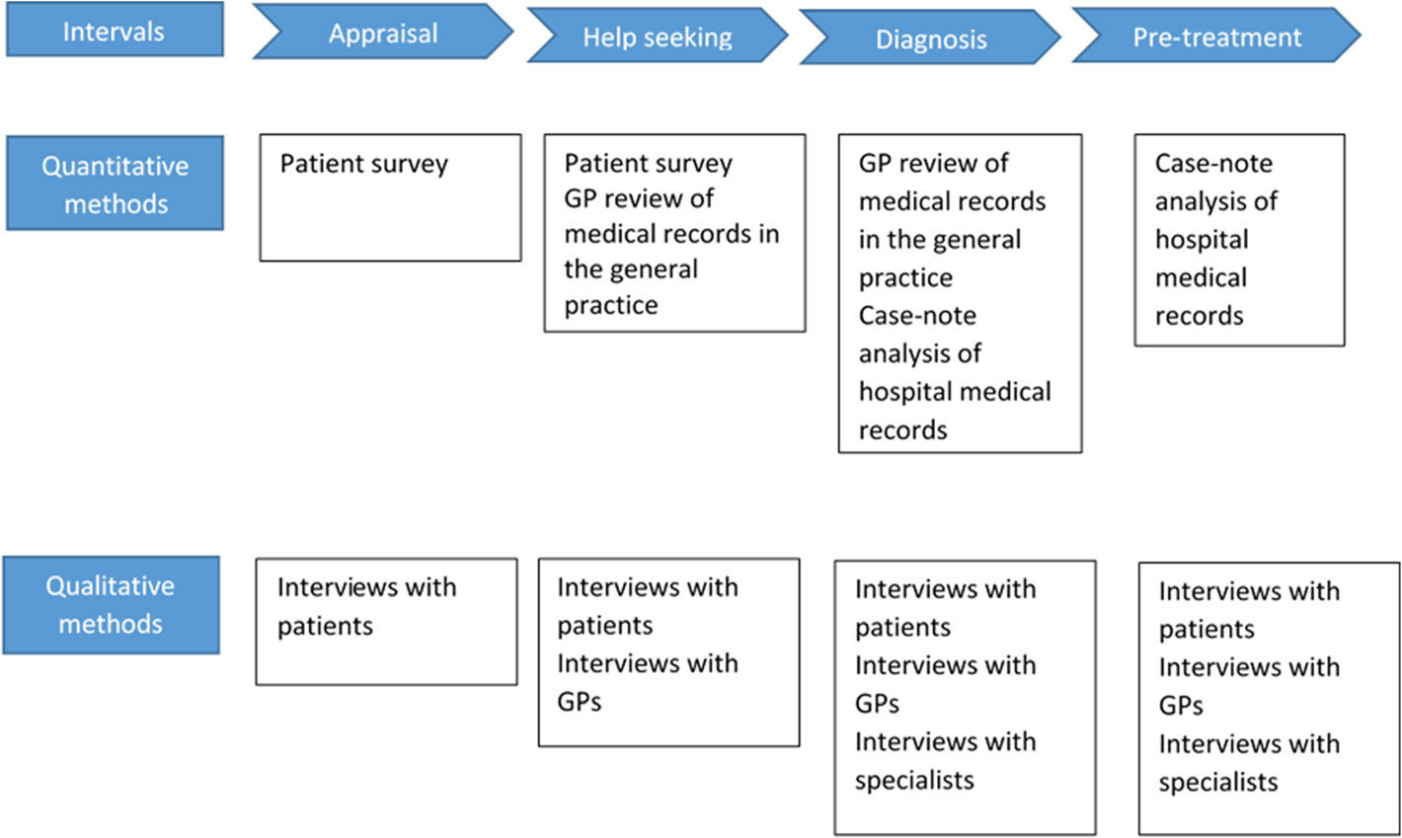
Study design of the LEAD. GP: General Practitioner

LEAD is a prospective, observational cohort study using a mixed-method approach to gather and interpret quantitative and qualitative data. Quantitative information on time intervals (see Fig. 2) and other factors will be gathered via patient survey, GP review of general practice records, and case-note analysis of hospital records. Qualitative information will be gathered via structured interviews with lung cancer patients, general practitioners (GPs), and hospital specialists.

The LEAD project will be conducted in five sites across three states in Australia: three Integrated Cancer Services in Melbourne, Victoria; one public hospital in Sydney, New South Wales; and, one public hospital in Brisbane, Queensland. These health services provide coverage for all of metropolitan regions of Melbourne, Sydney and Brisbane and include significant numbers of lung cancer patients, including CALD patients.

### Participants and recruitment

Three groups of participants will be recruited for this study: lung cancer patients, GPs and hospital specialists.

#### Lung cancer patients

Lung cancer patients will be involved in both the quantitative and the qualitative components of LEAD. The quantitative component involves a patient survey and a case-note analysis of hospital records. The qualitative component involves an interview. The patient eligibility criteria are: (1) have a diagnosis of primary lung cancer at the study sites within the past month or during the recruitment phase, and (2) be of CALD or Anglo-Australian descent. We will use prospective recruitment and also include patients who have been diagnosed within the past month to minimise the risk of recall bias and participant attrition due to death or terminal illness.

Patients of CALD descent are defined in the study as those who were born overseas and from one of the following ethnic groups: Arabic, Chinese, Greek, Italian, and Vietnamese. These are the most common ethnic groups for overseas-born people in Australia [15]. Anglo-Australian patients will be defined as those who were born in Australia or other major English-speaking countries (Canada, New Zealand, the United Kingdom, and the US). Patients who are pregnant or aged under 18 years will be excluded from the study because lung cancer among these two groups is very uncommon and those patients tend to have a different diagnostic pathway to the general population [17, 18].

Eligible patients will be identified through the list of new cases presented at the respective lung multidisciplinary team meetings. Additional recruitment sources, such as the bronchoscopy lists, might be used for some study sites on a local basis. The project coordinators will regularly go through these lists throughout the recruitment phase or until the required sample size has been reached.

After an eligible patient has been identified, the site coordinator will send a letter to invite the patient to participate in the patient survey and the patient interview. Patients may consent to take part in either or both activities. A waiver of consent has been obtained for the case-note analysis of hospital records, and the required data will be gathered by a hospital staff member with authorised access to medical records.

The invitation letter will be sent together with the patient survey and a reply-paid envelope. Two weeks after the initial invitation, the patients will receive a reminder phone call and a reminder letter from the site coordinator. For CALD patients, the invitation letter and the survey will be provided in English as well as their preferred languages. As an incentive to take part in the interview, the patients will be offered a $40 gift card, in line with the average hourly Australian wage.

#### GPs

**Fig. 3:**
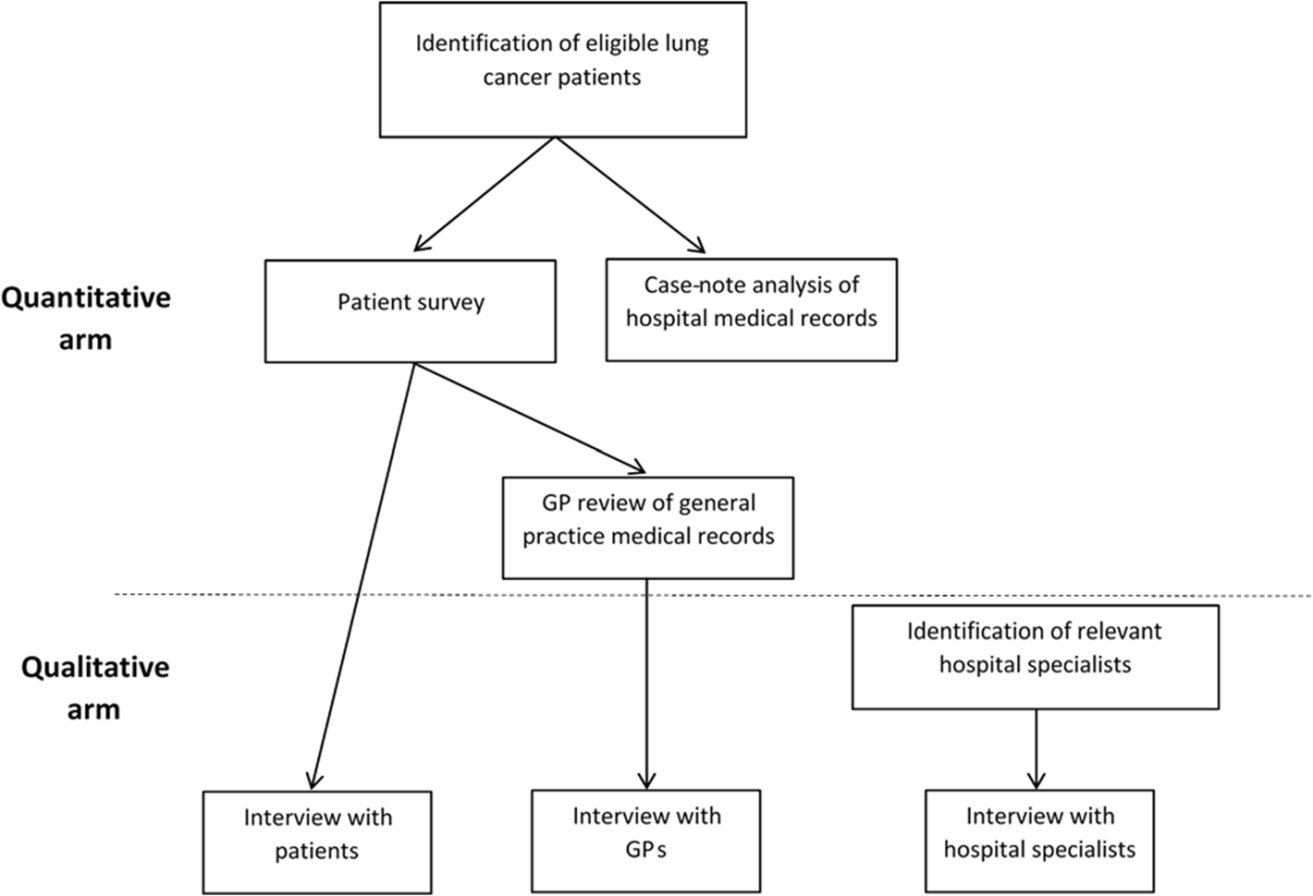
Flow chart of the LEAD project. GP: General Practitioner

The GP of enrolled lung cancer patients will be invited to take part in LEAD. Their involvement in the quantitative component will be in the form of a review of their general practice records, and their involvement in the qualitative component will be in the form of an interview. The GP will be identified by the patients who have chosen to participate in the study and who have provided consent for the research team to access their hospital and general practice medical records (see Fig. 3).

With their patients’ consent, the GPs will be posted a letter inviting them to complete a review of the patient’s medical records at the general practice and to take part in an interview. The letter will be posted together with a GP review proforma, the patient’s consent form, and a reply-paid envelope. A reminder letter will be sent 2 weeks after the initial invitation. To increase GPs’ interest in taking part in the study, a certificate of participation will be provided to GPs who complete the review. The GPs will be able to use this certificate to self-report to relevant medical colleges for Continuing Professional Development points. As an incentive to take part in the interview, the GPs will be offered a $200 gift card, In line with the average hourly consultancy rate for a GP and for lost earnings during the interview.

#### Hospital specialists

Hospital specialists at the five study sites providing care to lung cancer patients will be invited to take part in a qualitative interview. Hospital specialists will include thoracic surgeons, respiratory physicians, medical oncologists, radiation oncologists, nurses, radiologists, pathologists, palliative care physicians, social workers, and allied health professionals. The LEAD coordinators at each site will send an invitation email to these staff and ask interested staff to contact the project manager directly. A reminder email will be sent 2 weeks after the initial invitation. No financial incentive payment will be provided to the specialists as the interviews will be conducted during their normal working hours at the study sites.

### Data collection and measures

#### Patient Survey

The patient survey comprises the Cancer Symptom Interval Measure (C-SIM) used in previous lung cancer studies [19, 20]. It includes questions on the timing of the onset and presentation of symptoms potentially related to lung cancer, as well as questions about GP-initiated tests and the patient’s socio-demographic characteristics (e.g. education, occupation), health status (e.g. smoking history and co-morbidities), and health literacy. When completing the survey, the patients will be able to choose: 1) to complete the survey anonymously, or (2) to provide identifiable personal information and written consent for the research team to access their hospital and general practice medical records.

#### GP review proforma

GP review proforma is based on an earlier one used by J Emery, F Walter, V Gray, C Sinclair, D Howting, M Bulsara, C Bulsara, A Webster, K Auret, C Saunders, et al. [20]. It captures key data on presentations to general practice and investigations conducted by GPs prior to referring the patient to a specialist. It will also collect demographic and health system information of the GP’s practice.

#### Case-note analysis tool

Data for the case-note analysis will be collected using an audit tool with identifiable patient information removed. This audit tool is based on those previously used by the research team [21, 22] and will collect data relevant to lung cancer diagnosis and treatment (e.g. date of diagnosis, date of GP referral) and patients’ demographic background (e.g. gender, age).

#### Qualitative interviews

Questions for the interviews with lung cancer patients, GPs, and hospital specialists will be developed from the Model of Pathways to Treatment, and cover patient, health provider, and health system factors that might influence the diagnostic pathway of lung cancer patients. For interviews with lung cancer patients, questions will also be based on the interview schedule used in an earlier study [20]. With participants’ consent, all interviews will be audio-taped.

Lung cancer patients will be able to choose to have the interview conducted face-to-face or via telephone. The interview will be conducted at a time convenient to the participant and will last approximately 1 hour. For CALD patients, a qualified interpreter or a bilingual researcher will be involved in the interview as required. The patient’s carer is welcome to take part in the interview if preferred by the patient. For GPs and hospital specialists, the interviews will be conducted via telephone at a time convenient to the participant. The interview will last between 30 min to 1 hour.

### Ethics, consent and permissions

Our project has received ethics approval for a multiple-site study from the Monash Health Human Research Ethics Committee (HREC/16/MonH/311) and research governance approval from all participating sites. Three forms of consent will be used in the study: waiver of consent, implied consent and written consent. Waiver of consent will be used for the case-note analysis component and implied consent will be used for the patient survey and the GP review components. Written consent will be obtained for patients who have provided consent in the survey for the research team to access their hospital and general practice medical records. It will also be used for all participants in the qualitative arm.

### Statistical Considerations

#### Quantitative component

Comparions between CALD and Anglo-Australian patients on time intervals will be performed using the logrank test. Cox proportional hazards regression will be used to estimate the relative effect of factors (e.g. patient factors, healthcare provider factors, health systems factors) on the underlying hazard rate governing time intervals. Independent groups will be compared using t-tests for continuous variables and chi-square tests for categorical variables. Linear modelling methods for continuous and categorical data may also be used to undertake comparisons adjusted for selected covariates.

A Danish cohort study demonstrated that 60 days was a clinically significant diagnostic interval beyond which mortality increased [23] while another study reported that the median tumour volume doubling time of all lung cancers is 98 days (IQR 108 days) [24]. Based on these data, a 20% increase in tumour size every 28 days appears plausible and capable of affecting disease staging. A between-group difference in the time to treatment of 28 days or more would therefore be clinically significant. A total of 290 participants (145 per group) will provide 90% power with a two-sided alpha of 0.05 to detect an absolute difference of 28 days in median time to diagnosis (60 days versus 88) based on a log-rank test. We anticipate that 724 patients (362 per group) will need to be contacted in order to obtain a sample of this size assuming a 50% patient survey completion rate (based on previous studies, e.g., Emery et al., 2013; Walter et al., 2015), and a 80% GP review completion rate.

#### Qualitative component

Based on our experience, data saturation is likely to be reached with the following interview sample sizes: 60 lung cancer patients (10 patients per language group), 20 GPs, and 20 specialists.

All interviews will be audio-taped, and transcription, translation, coding, and analysis will occur concurrently with data collection. Thematic analysis will be conducted using a constant comparative method to identify similarities and differences in the content [25]. Data will be analysed inductively and deductively. The interviews will then be incorporated into NVivo version 10 for more structured coding and analysis.

## Discussion

The LEAD project is the first Australian study to compare the time intervals along the lung cancer diagnostic pathway between CALD and Anglo-Australian patients. The project will also explore the underlying patient, healthcare provider, and health system factors that influence the time intervals in the two groups. This information will improve our understanding of the effect of cultural diversity on health outcomes among lung cancer patients and will inform future interventions aimed at early diagnosis and treatment for lung cancer, particularly patients from CALD backgrounds.

There are a number of strengths in the design of the LEAD study that could be considered in future studies. Firstly, the Model of Pathways to Treatment (Walter et al., 2012) will be used to conceptualise and measure the various stages along the cancer diagnostic pathway. This model provides clear definitions and measurements of time intervals along the cancer diagnostic pathway and has been incorporated into the Aarhus Statement, an international guideline for the design and reporting of studies on early cancer diagnosis [6]. The adoption of such a model enables a systematic approach in data collection and allows data comparison between studies and across cancer types.

Secondly, the study will use both quantitative and qualitative methods and will collect information from multiple groups, including lung cancer patients, GPs and specialists. The inclusion of these different methods and participant groups is particularly important given the complexity of the cancer diagnostic and pre-treatment pathway. Compared to earlier studies where a single research method or participant group was used [e.g. 10, 13], such an approach enables a more comprehensive picture of the diagnostic pathway.

Thirdly, the study includes both CALD and Anglo-Australian patients. As noted in a systematic review of cancer beliefs in CALD populations, one limitation of current studies in this area is that few have comparator groups of the local population, making it difficult to disentangle local- versus ethnicity-specific factors in these studies [14]. Compared to these studies, the inclusion of both groups in our study enables a direct comparison between the two groups, leading to a deeper understanding of cultural differences in the diagnostic pathways.

In conclusion, the LEAD project will be the first Australian study to provide comprehensive data on the lung cancer diagnostic pathway for CALD and Anglo-Australian patients. Guided by the Model of Pathways to Treatment, this mixed-method, observational cohort study will inform the development of interventions aimed at improving the diagnosis and treatment of lung cancer in multicultural countries.

## Abbreviations

CALD: Culturally and linguistically diverse
C-SIM: Cancer Symptom Interval Measure
GP: General Practitioner
LEAD: Lung cancer diagnostic and treatment pathways: a comparison between Culturally and linguistically diverse (CALD) and Anglo-Australian patients
US: United States
